# eIF4E phosphorylation mediated LPS induced depressive-like behaviors via ameliorated neuroinflammation and dendritic loss

**DOI:** 10.1038/s41398-023-02646-5

**Published:** 2023-11-17

**Authors:** Qichao Gong, Weifen Li, Tahir Ali, Yue Hu, Shengnan Mou, Zizhen Liu, Chengyou Zheng, Ruyan Gao, Axiang Li, Tao Li, Ningning Li, Zhijian Yu, Shupeng Li

**Affiliations:** 1 State Key Laboratory of Chemical Oncogenomics, Peking University Shenzhen Graduate School, Shenzhen, 518055 Guangdong, China; 2 Department of Infectious Diseases, Shenzhen Key Laboratory for Endogenous Infections, The 6th Affiliated Hospital of Shenzhen University Health Science Center. No 89, Taoyuan Road, Nanshan District, 518052 Shenzhen, China; 3 College of Forensic Medicine, Institute of Forensic Injury, Xi’an Jiaotong University Health Science Center, Xi’an, Shaanxi China; 4 Tomas Lindahl Nobel Laureate Laboratory, Precision Medicine Research Centre, The Seventh Affiliated Hospital of Sun Yat-sen University, 518107 Shenzhen, China; 5 Institute of Chemical Biology, Shenzhen Bay Laboratory, 518132 Shenzhen, China; 6 Department of Psychiatry, University of Toronto, Toronto, ON Canada

**Keywords:** Neuroscience, Molecular neuroscience

## Abstract

The translational defect has emerged as a common feature of neurological disorders. Studies have suggested that alterations between opposing and balanced synaptic protein synthesis and turnover processes could lead to synaptic abnormalities, followed by depressive symptoms. Further studies link this phenomenon with eIF4E and TrkB/BDNF signaling. However, the interplay between the eIF4E and TrkB/BDNF signaling in the presence of neuroinflammation is yet to be explored. To illuminate the role of eIF4E activities within LPS-induced neuroinflammation and depression symptomology, we applied animal behavioral, biochemical, and pharmacological approaches. In addition, we sought to determine whether eIF4E dysregulated activities correlate with synaptic protein loss via the TrkB/BDNF pathway. Our results showed that LPS administration induced depressive-like behaviors, accompanied by neuroinflammation, reduced spine numbers, and synaptic protein dysregulation. Concurrently, LPS treatment enhanced eIF4E phosphorylation and TrkB/BDNF signaling defects. However, eFT508 treatment rescued the LPS-elicited neuroinflammation and depressive behaviors, as well as altered eIF4E phosphorylation, synaptic protein expression, and TrkB/BDNF signaling. The causal relation of eIF4E with BDNF signaling was further explored with TrkB antagonist K252a, which could reverse the effects of eFT508, validating the interplay between the eIF4E and TrkB/BDNF signaling in regulating depressive behaviors associated with neuroinflammation via synaptic protein translational regulation. In conclusion, our results support the involvement of eIF4E-associated translational dysregulation in synaptic protein loss via TrkB/BDNF signaling, eventually leading to depressiven-like behaviors upon inflammation-linked stress.

## Introduction

Major depressive disorder (MDD) is a severe psychiatric disease with increasing prevalence globally and affects human individuals in different life domains [1, 2]. Emerging evidence amply supports that non-neuronal cells, including glial cells (inflammatory milieu), participate in the neurobiology of depression, as dysregulated glial cell expression/activation has been reported in depression subjects and animal models under different stimuli (stress) [3, 4]. Mechanistically, gliosis and hyper-inflammation elevate pro-cytokines levels, accompanied by reactive oxygen species, contributing to neuronal damage and mood and behavior alteration [5, 6]. As we reported previously, LPS administration led to dysregulated microglia and astrocyte activities/expression in the brains of experimental mice and could be rescued by antidepressants, including fluoxetine and melatonin [7, 8]. Mechanistically, LPS-induced neuroinflammation-associated depression could be linked to synaptic protein altered expression via PI3K/Akt signaling, which can further regulate mTOR signaling via growth factors or neurotransmitters [9–12].

The accumulated evidence, including ours, shows protein synthesis is dysregulated in response to stimuli and contributes to altered synaptogenesis, which plays a crucial role in CNS disorders, including depression [13–15]. This multi-step reaction/process of protein synthesis is regulated by numerous other regulatory factors, including translational initiation and elongation factors. eIF4E (eukaryotic translation initiation factor 4E) is the cap-binding protein that binds to mRNA and regulates the recruitment of ribosomes and translation initiation. However, the regulatory function of eIF4E is tightly regulated at the post-translational level by phosphorylation and inhibitory protein bindings [16, 17]. MAP kinases ERK1/2 and P38 could modulate mRNA translation via MNK1/2 [15, 18, 19], which phosphorylate eIF4E (at Ser209), followed by an increased subset of mRNAs contributing to memory formation and circadian rhythms regulation. Further, MAP kinase signaling inhibition could enhance depressive symptoms and block antidepressant activities [20]. Similarly, P38 activation participates in depression symptomology as an immune mediator [21].

Previous studies show that mice ablation of eIF4E phosphorylation demonstrated concomitant depression or anxiety-like behaviors. Moreover, eIF4E phosphorylation is required for the anti-depressive effects of fluoxetine, possibly via regulating the translation of a subset of mRNAs linked to inflammation, the extracellular matrix, pituitary hormones, and the serotonin pathway [20]. To further address the underlying mechanisms of eIF4E phosphorylation in the etiology of depression, we showed that eFT508 treatment significantly rescued LPS-induced depressive-like behaviors, neuroinflammation, and synaptic protein dysregulations in the mice. eFT508 is a selective inhibitor of MNK1/2, indirectly regulates eIF4E [22, 23]. Moreover, K252a (TrkB/BDNF inhibitor) treatment could ablate the anti-depressive and anti-inflammatory activities of eFT508, suggesting the regulatory role of eIF4E in the synaptic processes via the TrkB/BDNF signaling during depression development.

## Methods

### Animals

Adult C57 BL/6J male mice weighing 20–22 g (age 6–8 weeks) were purchased from Guangdong Medical Laboratory Animal Center, China. The experimental animals were housed (*n* = 4/cage) at the Laboratory Animal Research Center, Peking University Shenzhen Graduate School, under a 12 h light/12 h dark cycle at 18–22 °C. They had free access to food and tap water throughout the study. The experimental procedures were designed to minimize animal suffering. All experimental procedures were performed according to the protocols approved (Approval Number: 11110) by the Institutional Animal Care and Use Committee of Peking University Shenzhen Graduate School.

### Drugs and reagents

The eFT508 (CAS No. 1849590-01-7) used in this study was purchased from Shanghai Send Pharm, prepared for in vivo dosing, and diluted in 0.9% saline. The eFT508 dosing was selected according to the previous reports [22, 23].

The lipopolysaccharide (LPS) was purchased from Sigma-Aldrich. The K252a was purchased from Med Chem Express (MCE) and diluted in 0.1% DMSO.

### Experimental design for drug treatment

The study was conducted in two experiments.

In the first experiment, animals were divided into three groups; each group included 8–10 mice, and repeat each experiment with three batches of mice in the meanwhile. The group names are as follows: normal saline-treated (NC), LPS (1 mg/kg, once a day, i.p.) treated (LPS), and LPS + eFT508 (5 mg/kg, once a day, i.p.).

The second experiment was planned to explore the TrkB/BDNF role in LPS-induced depression, and mice were treated with a TrkB inhibitor (K252a). The animals were divided into three groups; each group included 10–12 mice, and repeat each experiment with two batches of mice. The animal groups were LPS-treated, LPS + eFT508, and LPS + eFT508 + K252a (25 μg/kg, once a day, i.p.).

The same treatment for animals and behavior analysis was repeated three times under similar conditions. For the behavior test, the first batches for SPT, the second for TST and OFT, and the third batch of mice were used for FST and OFT behavior tests. For other experiments, the sample from the first batch we used for Western blotting and ELISA, the sample from the second batch was used for staining and the third batch we used for qPCR. All behavior tests were performed 24 h after the last LPS injection.

After 24 h of the last LPS injection, mice were sacrificed after behavior analysis. Plasma and brain tissues were collected and stored at freezing temperatures (−80 °C) until further investigation.

### Behavior analysis

#### Open field test (OFT)

OFT was performed according to the previously developed protocols [24]. Briefly, mice were adapted to the experimental room for one hour and placed in the 45 cm × 45 cm × 30 cm chamber. A total of 5 min of video was recorded to observe the mice’s locomotor activity. The total distance covered by mice was measured, analyzed, and expressed in meters.

#### Sucrose preference test (SPT)

A sucrose preference test was performed while using a two-bottle free-choice paradigm. To assess the individual sucrose intake, mice were deprived of water and food for 24 h on the third day after LPS administration. Each mouse had free access to two sucrose and water bottles the next day. The position of water and sucrose-containing bottles weres changed after 2.5 h. Finally, the volume of consumed water and sucrose solution were recorded and calculated by the following formula:

$${{\rm {Sucrose}}}\,Preference=\frac{{{\rm {Sucrose}}}\,{{\rm {consumption}}}}{{{\rm {Water}}}\,{{\rm {and}}}\,{{\rm {sucrose}}}\,{{\rm {consumption}}}}\times 100 \%$$

#### Forced swimming test (FST)

The forced swimming test (FST) was performed according to previously developed protocols [25]. The experimental animals were trained for swimming, and pre-experiment FST was performed to select healthy and normal mice. To perform the FST, the animals were placed in a Plexiglas cylinder (height: 70 cm, diameter: 30 cm) filled with water over the 30 cm level at a temperature of 23 ± 1 °C. The video was taped for 6 min, and the last 5 min were blindly analyzed. Mice were considered immobile when they remained floating motionless in the water and just making a move to keep their nose above the water’s surface. The horizontal movement of the animals throughout the cylinder was defined as swimming, while vertical movement against the cylinder wall was defined as climbing. EthoVision XT software was used to record the video and analysis.

#### Tail suspension test (TST)

The tail suspension test was performed as described previously [24, 26]. Briefly, the mice were individually suspended about 40 cm above the floor by their tail with the tape in the rectangular compartment (55 cm height × 20 cm width × 11.5 cm depth). A total of 5 min of video were recorded and analyzed. EthoVision XT software was used to record and analyze the data.

#### Grip strength test (GST)

The grip strength of paws was assessed to measure forelimb muscle power. Each animal was placed on the central table of the grip plate, and the tail was pulled gently to encourage the mouse to grasp the grip plate. When the mouse had firmly held the grip plate, the tail was pulled and moved till the animal relinquished its grip. The maximum gripping power was recorded. The experiment was repeated thrice per animal, and the evaluation value was the most significant value among the three results.

#### Hanging test (HT)

An inverted grid suspension experiment was used to evaluate the gripping power of the mouse limbs. Each animal was placed in the center of a 21 cm × 21 cm wire grid (line width of ~0.1 cm, spacing, 0.5 cm). The grid was then tapped to make the mouse grip tight, following which the grid was slowly inverted to horizontal. The time the mouse hung onto the grid (grip time) was recorded. The experiment was repeated thrice per animal, and each mouse’s average hanging time value was calculated as the evaluation value. The 90 s (or longer) was set as the cut-off value.

#### Pole test (PT)

The lever climbing experiment was used to evaluate the motor and coordination abilities of the limbs of mice. A self-made wooden pole with a length of about 50 cm and a diameter of about 1 cm is wrapped with gauze to increase friction. The wooden bar was placed horizontally on the table, and the mouse was placed head down on top of the appearance. The mouse crawled autonomously under the drive of no external force, and the time for the mouse to climb from the top of the pole to the platform at the bottom was recorded (climbing time).

### Nitric oxides and H_2_O_2_ measurement

The levels of Nitric oxides and H_2_O_2_ were analyzed using commercially available kits (Beyotime Institute of Biotechnology, China, CAT# S0021M, and CAT# S0038, respectively) [27, 28]. Briefly, for the NO detection, 2 μL of the sample (serum/homogenates) were added to the mixture of Reagents (Gryess Reagent) R1 + R2 (50 μL + 50 μL), and absorbance was recorded at 540 nm. Similarly, for the H_2_O_2_ detection, a 2 μL sample (serum/homogenates) was added to the 100 μL of the detection reagent, and after 30 min incubation at 37 °C, the absorbance was recorded at 560 nm.

### ELISA

The frozen hippocampal and cortical tissue was lysed with RIPA buffer and homogenized on ice. Supernatants were collected after centrifugation and stored at freezing temperature for further analysis. According to the manufacturer’s protocols, the expression of cytokines was quantified using ELISA kits (mlbio). Briefly, 50 µL standard/sample and 100 μL streptavidin-HRP were added and incubated for 1 h at 37 °C. The dish was then washed, and reaction substrates A and B were added to each well. The plate was incubated for 15 min at 37 °C. Finally, the reaction was stopped, and the optical density was measured accordingly.

### Immunofluorescence

Immunofluorescence staining was performed according to previously reported protocols [29]. Brain tissue sections (30 µm thick) were briefly washed with PBS for 15 min (5 min × 3). After washing, the slides were treated with blocking buffer (10% goat serum in 0.3% Triton X-100 in PBS) for 1 h at room temperature. After blocking, the tissue was treated with primary antibodies (IBA-, GFAP, and NeuN) overnight at 4 °C. The next day, secondary antibodies (Alexa Flour secondary antibodies, Thermo Fisher) were applied at room temperature for 1 h. The sections were washed with PBS for 5 min three times. After washing, sections were transferred to slides, and glass coverslips were mounted using the mounting medium. The images were taken under a Nikon A1R Laser scanning confocal microscope.

### Quantitative real-time PCR analysis

Briefly, total RNA was extracted from hippocampus tissues with TRIzol reagent (Invitrogen), and cDNA was synthesized using TransScript One-Step gDNA Removal and cDNA Synthesis SuperMix Kit (Transgen Biotech). qRT-PCR was performed using TransScript Tip Green qPCR SuperMix (Transgen Biotech) according to the manufacturer’s instructions, with each sample run at least in duplicate. Relative transcript abundance was normalized to GAPDH.

### Golgi staining

The FD Rapid Golgi Stain Kit (FD Neuro Technologies, Ellicott City, MD) was used to perform Golgi staining. Briefly, after removing, the animal brain was rinsed quickly in double-distilled water, immersed in impregnation solutions (A/B) (5 ml solution for each tissue), and stored at room temperature for 2 weeks. The brain tissues were transferred to solution C and stored for 72 h (the solution was replaced after 24 h), followed by freezing. Afterward, 100- to 200-μm sections were prepared using a sliding microtome and mounted to gelatine-coated microscope slides. Then the brain tissue was placed in a staining solution for 10 min, rinsed with double distilled water, followed by dehydration (sequential rinse 50%, 75%, and 95% ethanol) and xylene treatment, and finally examined under an inverted fluorescence microscope IX73 Olympus.

### Western blotting

According to the developed protocols, western blotting was performed. Briefly, Denatured samples (boiled at 100 °C for 10 min) were separated on SDS–PAGE and then transferred to the nitrocellulose membrane. The membrane was blocked with non-fat milk in TBST (Tris-buffered saline, 0.1% Tween 20), then incubated in primary antibody (1: 500; 1:1000) overnight at 4 °C. The next day, the membrane was treated with a secondary antibody (1:500) for 1 h at room temperature. For detection, the ECL Super signal chemiluminescence kit was used according to the manufacturer’s protocol. Blots were developed using Chemidoc mp Bio-red. The densitometry analysis of the bands was performed using image lab software.

### Statistical analysis

Western blot bands and morphological data were analyzed using ImageJ and image lab software (Image J 1.30), SPSS. Sample size selection was made as described previously [29, 30]. Data were presented as mean ± SEM. One-way ANOVA followed posthoc Tukey Multiple Comparison tests to compare different groups. *p* < 0.05 was regarded as significant. *p < 0.05, **p < 0.01, ***p < 0.001, ****p < 0.0001.

## Results

### eFT508 treatment rescued depressive-like behaviors and prevented neuroinflammation

**Fig. 1:**
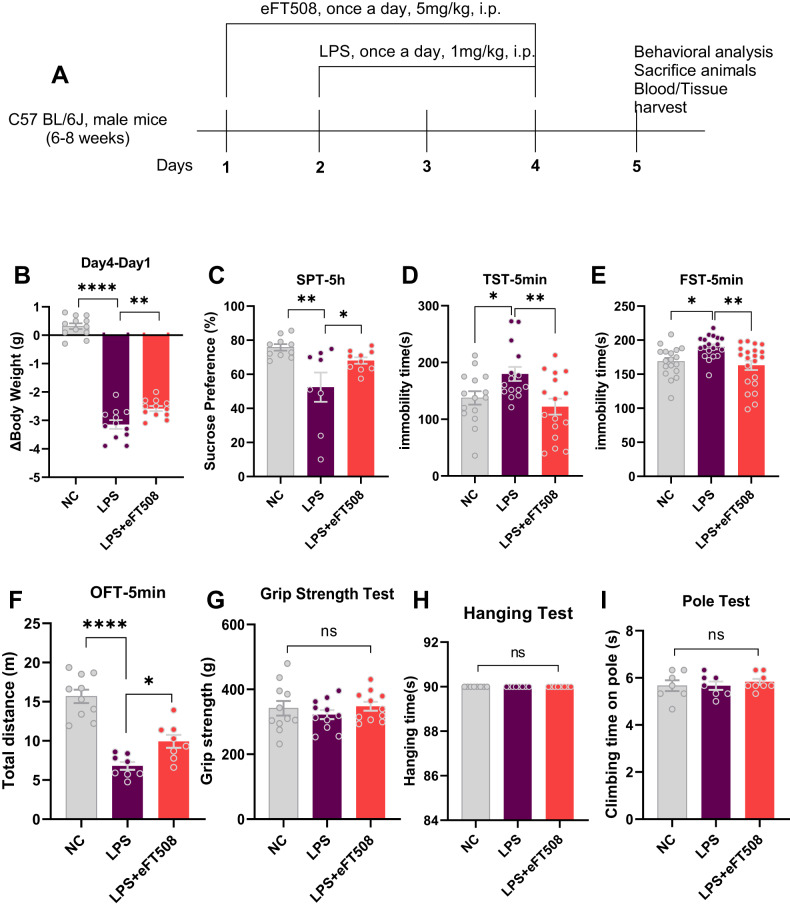
eFT508 reduced LPS-induced depressive-like behaviors. **A** Drug treatment schedule and experimental approach. **B** Relative body weight changes. **C** Sucrose preference test, *n* = 8–10. **D** Tail suspension test, *n* = 15–16. **E** Force swimming test, *n* = 18–21. **F** Open field test, *n* = 8–10. **G** Grip strength test, *n* = 11. **H** Hanging test and pole test. **I** *n* = 8. All the values are expressed as mean ± SEM, one-way ANOVA followed by Turkey’s multiple comparison tests. *p < 0.05, **p < 0.01, ***p < 0.001, ****p < 0.0001.

LPS is widely used as a depression agent via neuroinflammatory inducer [31, 32]. Here, we initially measured depressive-like behaviors after LPS administration. As shown in Fig. 1, LPS-treated mice displayed increased immobility and decreased sucrose preference during behavior tests (TST, FST, and SPT) and could be rescued by eFT508 treatment. In contrast, no significant changes in the mice’s performance could be found in HT, GSP, and PT tests after LPS and eFT508 administration.

**Fig. 2:**
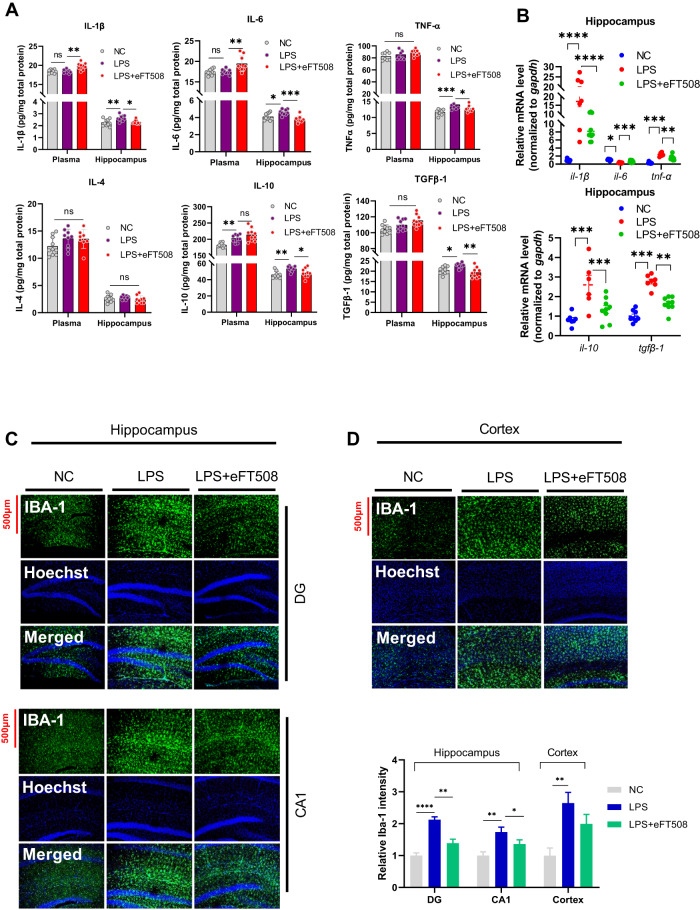
eFT508 attenuates LPS-induced dysregulation of cytokines and reduces IBA-1 expression in the hippocampus. **A** Changes of inflammatory cytokines in plasma and hippocampus. **B** Changes of inflammatory cytokines (mRNA level) in the hippocampus. **C** Representative immunofluorescence of IBA-1 in the hippocampus and cortex (**D**), with bar graphs showing relative IBA-1 intensity, *n* = 4. All values were expressed as mean ± SEM, one-way ANOVA followed by post hoc analysis. *p < 0.05, **p < 0.01, ***p < 0.001, ****p < 0.0001.

Previous results from ours and others showed that LPS-induced depressive-like behaviors are linked to neuroimmunomodulation [31, 32], including dysregulation of pro and anti-inflammatory cytokines. Figure 2 shows that increased IL-1β, IL-6, and IL-10 were found in the mice plasma after LPS administration. However, eFT508 treatment significantly reduced IL-1β, IL-6, IL-10, TNF-α, and TGFβ-1 expression in the hippocampus of the LPS-treated mice. No significant changes in IL-4 expression could be detected in the plasma and hippocampal tissues (Fig. 2A). Similarly, cytokines, including IL-1β, IL-6, TNF-α, IL-10, and TGFβ-1 mRNAs, were downregulated after eFT508 treatment (Fig. 2B). Decreased IBA-1 level was found in the hippocampus (DG, CA-1) and cortex of the eFT508-treated mice in the presence of LPS (Fig. 2C, D). However, the eFT508 treatment did not reduce the GFAP expression in the hippocampus but not in the cortex of eFT508-treated mice in the presence of LPS (Fig. S1). Further, no marked changes in the NeuN expression could be detected in the hippocampus and cortex experimental subjects (Fig. S2).

**Fig. 3:**
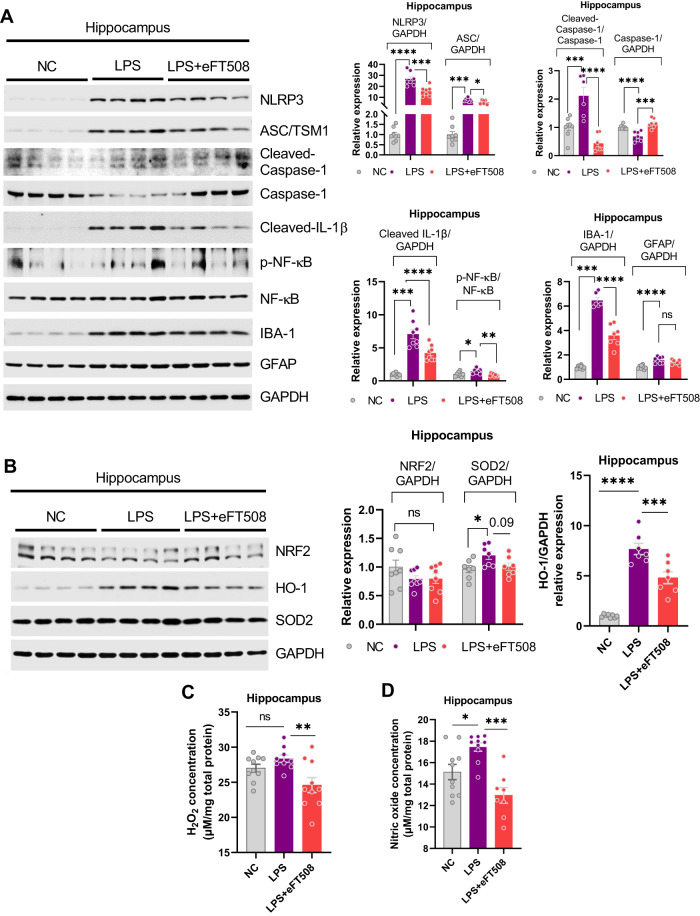
eFT508 treatment alleviates the inflammasome and redox signaling activation caused by LPS. **A** Representative western blot image with corresponding column graph showing NLRP3/ASC/Cleaved-Caspase-1/Caspase-1(pro)/Cleaved-IL-1β/NF-κB/IBA-1/GFAP expression. **B** Representative western blot images showing NRF2/HO-1/SOD2 expression, with corresponding column graph. **C** Representative quantitative graphs show the H_2_O_2_ and nitric oxide (**D**) levels in the hippocampus of experimental mice, *n* = 8–10. All the values are expressed as mean ± SEM, one-way ANOVA followed by Turkey’s multiple comparison tests. *p < 0.05, **p < 0.01, ***p < 0.001, ****p < 0.0001.

**Fig. 4:**
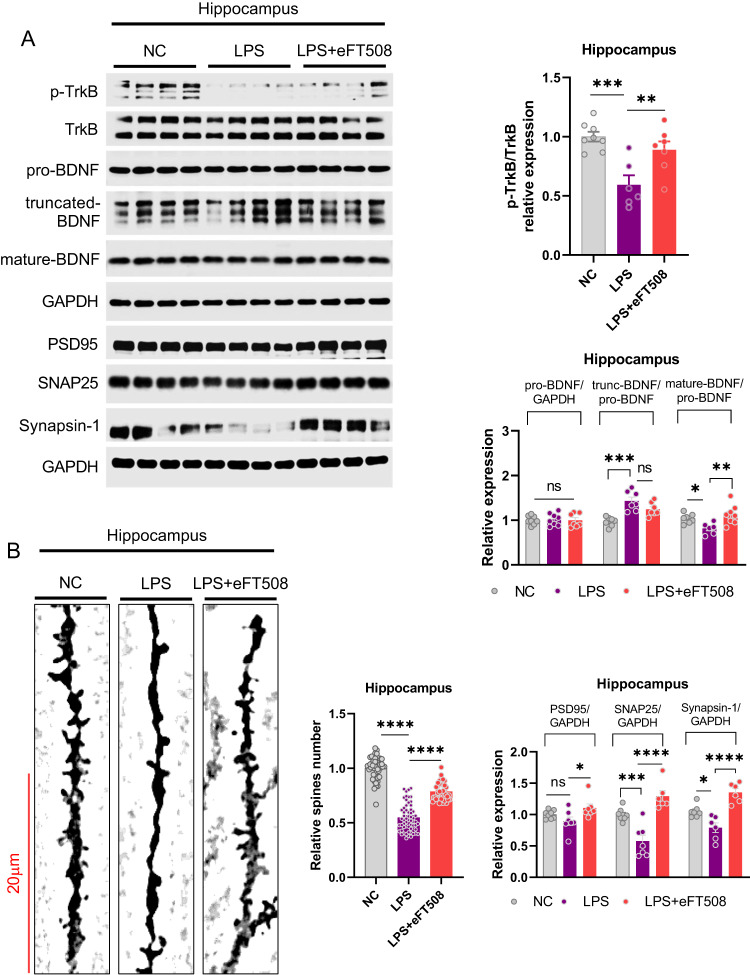
eFT508 treatment rescued LPS-induced synaptic defects. **A** Representative western blot images with corresponding column graph showing TrkB/pro-BDNF/truncated-BDNF/mature-BDNF and PSD95/SNAP25/Synapsin-1 expression (*n* = 6–8). **B** Golgi staining shows spine density, with the corresponding column graph showing the relative number of spines (*n* = 6–8). All the values are expressed as mean ± SEM, one-way ANOVA followed by Turkey’s multiple comparison tests. *p < 0.05, **p < 0.01, ***p < 0.001, ****p < 0.0001.

To further explore the molecular mechanisms underlying these findings, signaling pathways involved in the LPS-induced neuroinflammation were measured. eFT508 administration significantly reversed LPS-altered signaling molecules, including NLRP3, caspase-1 (cleaved), p-NF-кB, IBA-1, GFAP, and ASC/TSM1 expression (Fig. 3A). As it is well known that neuroinflammation and oxidative stress contribute parallelly to the etiology of depression symptoms, oxidative stress-related molecules were also measured, including NRF2, HO-1, SOD2, H_2_O_2_, and NO levels. As shown in Fig. 4, eFT508 reduced H_2_O_2_/NO and HO-1 levels, but it did not change NRF2 and SOD2 expression in the hippocampal tissue of experimental subjects (Fig. 3B–D).

Next, we sought to determine molecular signaling alterations involved in neuroinflammation-associated depression pathogenesis. Contrary to p-AMPKα and p-GSK3β, a significant decrease in phosphorylation of PI3K, Akt, MEK, ERK, P38, and eIF4E was found in eFT508-treated mice. However, no significant changes in ATF-6, IRE1α, p-eEF2, p-eIF2α, and mTOR were detected in the hippocampus of mice treated with eFT508 (Fig. S3). Overall, the findings suggested that decreased phosphorylation of eIF4E via MNK antagonist could prevent neuroinflammation-linked depressive-like behaviors.

### eFT508 treatment rescued LPS-induced synaptic defects

Synaptic defects are proposed as one of the most common and associated consequences of depression symptomology [33]. Thus, we measured synaptic signaling changes associated with the role of eIF4E against LPS-induced depression. Interestingly, eFT508 treatment significantly rescued LPS-altered expression of p-TrkB and BDNF (mature). Moreover, LPS reduced synaptic protein changes, including PSD95, SNAP25, and Synapsin-1, and dendritic spine reductions were largely recovered after eFT508 administration (Fig. 4). These results demonstrated a significant contribution of eIF4E in synaptic protein and structure stabilization against LPS-induced stress.

### TrkB/BDNF antagonism reversed eFT508-induced effects

**Fig. 5:**
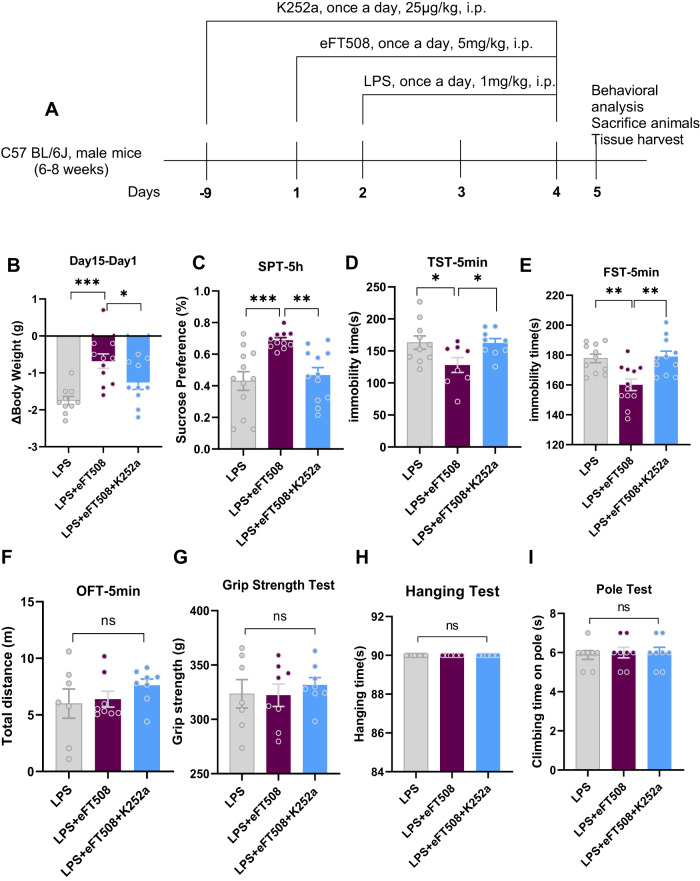
K252a treatment reversed the anti-depressive activity of eFT508. **A** Drug treatment schedule and experimental approach. **B** Relative body weight changes. **C** Sucrose preference test. **D** Tail suspension test. **E** Force swimming test. **F** Open field test. **G** Grip strength test. **H** Hanging test and pole test (**I**). **A**–**E** *n* = 8–11, and **F**–**I** *n* = 8. All the values are expressed as mean ± SEM, one-way ANOVA followed by Turkey’s multiple comparison tests. *p < 0.05, **p < 0.01, ***p < 0.001, ****p < 0.0001.

To further delineate the molecular signaling changes of eIF4E and its association with TrkB/BDNF, mice were treated with TrkB/BDNF signaling antagonist (K252a) with/only eFT508. K252a treatment blocked the antidepressive-like effects of eFT508, as demonstrated by increased immobility (TST and FST) and reduced sucrose preference (SPT) of LPS-treated mice (Fig. 5A). Similarly, no significant changes in the other behavior tests were found, including OFT, GST, HT, and PT (Fig. 5B–I).

**Fig. 6:**
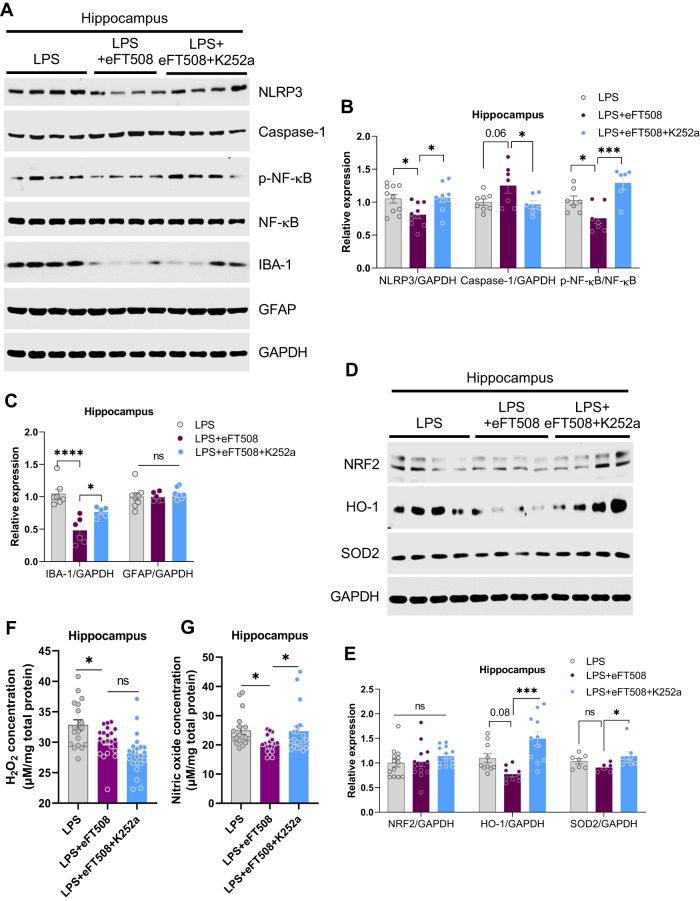
K252a treatment reversed the effects of eFT508 on alleviating inflammasome and redox signaling. **A** Representative western blot image showing NLRP3/Caspase-1(pro)/NF-κB/IBA-1/GFAP expression, with corresponding column graphs (**B**) and (**C**) (*n* = 6–8). **D** Representative western blot images showing NRF2/HO-1/SOD2 expression, with corresponding column graph (**E**), *n* = 8–10. **F** Representative quantitative graphs show the H_2_O_2_ and nitric oxide (**G**) levels in the hippocampus of experimental mice, *n* = 18–20. All the values are expressed as mean ± SEM, one-way ANOVA followed by Turkey’s multiple comparison tests. *p < 0.05, **p < 0.01, ***p < 0.001, ****p < 0.0001.

Next, we sought to determine whether TrkB/BDNF signaling antagonism had any effects on LPS-induced neuroinflammation and redox status. Notably, K252a treatment increased the expression of NLRP3, p-NF-kB, and IBA-1 in the eFT508-treated mice hippocampus in the presence of LPS (Fig. 6A–C). Furthermore, K252a treatment also elevated NO concentration and enhanced HO-1 and SOD2 expression; However, no changes in the H_2_O_2_ and NRF2 levels were detected after K252a treatment (Fig. 6D–G).

Further, K252a treatment reversed eFT508-induced p-AMPKα reduction while decreasing synaptic proteins of PSD95, SNAP25, Synapsin-1, and BDNF expression (Fig. S4). Additionally, K252a treatment increased the phosphorylation levels of eIF4E, mTOR, and Akt, while eEF2, PI3K, and ERK remained unchanged in the brains of the eFT508 and LPS-treated mice. However, K252a reduced MEK phosphorylation and altered cytokines levels in the brains of the experimental mice (Figs. S5 and S6). Overall, these findings indicated that eIF4E phosphorylation and activity are associated with neuroinflammation-linked depressive-like behaviors via the protein synthesis signaling pathway.

## Discussion

The present study demonstrated that LPS-induced neuroinflammation and depressive-like behaviors follow protein synthesis defects. At the molecular level, eIF4E dysregulation could significantly affect protein synthesis, leading to impaired cytokines and dendritic/synaptic protein expression. Moreover, the effects of eFT508 could be reversed by TrkB/BDNF antagonism, suggesting the critical role of eIF4E in the etiology of neuroinflammation-associated depression via TrkB/BDNF signaling.

Protein synthesis is a multi-step complex process, and an impaired protein translational stage has emerged as a common feature of various neuropsychiatric disorders [16, 34, 35]. Evidence shows that dysregulation of translation initiation factors, including eIF4E, could engender resistance to antidepressants like fluoxetine and neuroplasticity [20]. Similarly, defects in the protein translation factor, including eIF4E activity, could also coincide with the reduced activities of the NF-kB inhibitor [36, 37]. Previous preclinical and clinical results showed the causal link between inflammation and protein synthesis dysregulation in depressive conditions [38]. Similarly, in our findings, LPS-treatment induced protein synthesis defects via eIF4E dysregulations concurrent with neuroinflammation and depressive-like behaviors. Furthermore, increased oxidative/nitrosative stress upon LPS administration, which was reduced by eFT508 treatment, also supported the interplay between inflammation and oxidative stress and protein translational defects via eIF4E signaling. Besides, eFT508 treatment reduced AMPK activities by increasing its inhibitory phosphorylation sites, which may ultimately (indirectly) enhance eIF4E activities via MNK1 [39].

Synaptic proteins are tightly regulated by opposing processes of new protein synthesis and protein turnover in the neuron [40–42]. A defect that disturbs the balance between them could lead to neurological defects. Similarly, the translational regulatory factors are mainly regulated by two primary signalings, PI3K/Akt/mTOR and MEK/ERK kinase cascade. Activated mTOR and MEK/ERK kinase signaling induce eIF4E phosphorylation [43–45]. Besides, TrkB/BDNF signaling is also involved in synaptic protein synthesis and trafficking via PI3K and MAPK pathways [46, 47]. Former results show that TrkB/BDNF signaling contributes to spin morphogenesis, as glutamatergic synaptic activity stimulates BDNF and facilitates spine maturation via TrkB-PSD95 signaling [46–49]. Thus, TrkB/BDNF signaling alteration could probably lead to abnormal synaptic processes via protein translational factors, including eIF4E expression. In our findings, eFT508 treatment significantly recovered LPS-suppressed TrkB/BDNF signaling. It enhanced the spine numbers and synaptic protein expression, including PSD95 and Synapsin-1, supporting the interplay between eIF4E and TrkB/BDNF signaling in the regulation of synaptic protein processes and physiology.

In conclusion, LPS-induced neuroinflammation leads to depressive-like behaviors via eIF4E phosphorylation and may cause synaptic protein translational defects via TrkB/BDNF signaling.

### Supplementary information

Supplementary Figures

List of antibodies used in the study

## Data Availability

All data generated or analyzed during this study are included in this published article.
