# Supplementary material for: eIF4E phosphorylation mediated LPS induced depressive-like behaviors via ameliorated neuroinflammation and dendritic loss
Source: Transl Psychiatry. 2023 Nov 17;13:352. doi: 10.1038/s41398-023-02646-5 (PMC10656522; doi:10.1038/s41398-023-02646-5)
Supplement: Supplementary file 1 — Supplementary Figures [file 41398_2023_2646_MOESM1_ESM.docx]

**Figure S1. eFT508 treatment decreased the GFAP level in the cortex tissues of the LPS-treated mice instead of the hippocampus. (A)** Representative immunofluorescence of GFAP in the hippocampus and cortex **(B)**, with bar graphs showing relative GFAP intensity, n=4. All of the values were expressed as mean ± SEM, one-way ANOVA followed by post hoc analysis. **p* < 0.05, ***p* < 0.01, ****p* < 0.001,

**Fig.S2. LPS and eFT508 effects on NeuN expression. (A)** Representative immunofluorescence of NeuN in the hippocampus and cortex **(B)**, with bar graphs showing relative NeuN intensity, n=4. All of values were expressed as mean ± SEM, one-way ANOVA followed by post hoc analysis. *p < 0.05, **p < 0.01, ***p < 0.001, ****p < 0.0001.

*****p* < 0.0001.

**Figure S3. eFT508 treatment displayed an altered pattern of neuroinflammation-associated depression molecular signaling pathways.** Representative western blot images showing the expression level, including Phospho/total protein, of ATF6/IRE1α/AMPKα/eEF2/eIF2α/eIF4E/PI3K/Akt/mTOR/P38/MEK/ERK/GSK3β, n=6~8. All the values are expressed as mean ± SEM, one-way ANOVA followed by Turkey’s multiple comparison tests. **p* < 0.05, ***p* < 0.01, ****p* < 0.001, *****p* < 0.0001.

**Figure S4. K252a treatment reversed the neuroprotective effect of synaptic protein expression regulated by eFT508.** Representative western blot images showing the expression level of AMPKα/pro-BDNF/truncated-BDNF/mature-BDNF/PSD95/SNAP25/Synapsin-1, with corresponding column graph, n=8~12. All the values are expressed as mean ± SEM, one-way ANOVA followed by Turkey’s multiple comparison tests. **p* < 0.05, ***p* < 0.01, ****p* < 0.001, *****p* < 0.0001.

**Figure .S5. K252a treatment effects on the pattern of neuroinflammation-associated depression molecular signaling pathways.** Representative western blot images showing the expression level, including Phospho/total protein, of eEF2/eIF2α/eIF4E/PI3K/Akt/mTOR/P38/MEK/ERK, n=8~10. All the values are expressed as mean ± SEM, one-way ANOVA followed by Turkey’s multiple comparison tests. *p < 0.05, **p < 0.01, ***p < 0.001, ****p < 0.0001.

**Fig.S6. K252a treatment reversed the regulatory effects of eFT508 on the dysregulation of inflammatory cytokines.** Changes of inflammatory cytokines in the hippocampus, n=6~8. All of the values were expressed as mean ± SEM, one-way ANOVA followed by post hoc analysis. *p < 0.05, **p < 0.01, ***p < 0.001, ****p < 0.0001.
