# Supplementary material for: eIF4E phosphorylation mediated LPS induced depressive-like behaviors via ameliorated neuroinflammation and dendritic loss
Source: Transl Psychiatry. 2023 Nov 17;13:352. doi: 10.1038/s41398-023-02646-5 (PMC10656522; doi:10.1038/s41398-023-02646-5)
Supplement: Supplementary file 2 — List of antibodies used in the study [file 41398_2023_2646_MOESM2_ESM.docx]

Table 1. Detailed antibody information.

| Antibody | Company | Lot Number | Dilute | Source |
| --- | --- | --- | --- | --- |
| NLRP3 | Cell signaling technology | 15101 | 1/1000 | Rabbit |
| ASC/TSM | Cell signaling technology | 67824 | 1/1000 | Rabbit |
| Cleaved Caspase-1 | Cell signaling technology | 89332 | 1/1000 | Rabbit |
| Caspase-1 | proteintech | 22915-1-AP | 1/1000 | Rabbit |
| Cleaved-IL-1β | Cell signaling technology | 83186 | 1/1000 | Rabbit |
| p-NF-κB | Cell signaling technology | 3033 | 1/1000 | Rabbit |
| NF-κB | Cell signaling technology | 8242 | 1/1000 | Rabbit |
| Iba-1 | Cell signaling technology | 17198S | 1/1000 | Rabbit |
| GFAP | Cell signaling technology | 3670S | 1/1000 | Mouse |
| GAPDH | Cell signaling technology | 5174 | 1/1000 | Rabbit |
| ATF6 | Cell signaling technology | 65880 | 1/1000 | Rabbit |
| IRE1α | Cell signaling technology | 3294 | 1/1000 | Rabbit |
| p-AMPKα | Cell signaling technology | 2535 | 1/1000 | Rabbit |
| AMPKα | Cell signaling technology | 5832 | 1/1000 | Rabbit |
| p-TrkB | abcam | ab229908 | 1/1000 | Rabbit |
| TrkB | Cell signaling technology | 4603 | 1/1000 | Rabbit |
| BDNF | abcam | ab108319 | 1/1000 | Rabbit |
| p-eEF2 | Cell signaling technology | 2331 | 1/1000 | Rabbit |
| eEF2 | abcam | ab33523 | 1/1000 | Rabbit |
| p-eIF2α | Cell signaling technology | 3597 | 1/1000 | Rabbit |
| eIF2α | Cell signaling technology | 9722 | 1/1000 | Rabbit |
| p-eIF4E | Cell signaling technology | 9741 | 1/1000 | Rabbit |
| eIF4E | Cell signaling technology | 2067 | 1/1000 | Rabbit |
| PSD95 | abcam | ab18258 | 1/1000 | Rabbit |
| SNAP25 | abcam | ab41455 | 1/1000 | Rabbit |
| Synapsin-1 | Cell signaling technology | 25297 | 1/1000 | Rabbit |
| Nrf2 | Cell signaling technology | 12721 | 1/1000 | Rabbit |
| HO-1 | Cell signaling technology | 70081 | 1/1000 | Rabbit |
| SOD2 | Cell signaling technology | 13194 | 1/1000 | Rabbit |
| p-PI3K | Cell signaling technology | 4228 | 1/1000 | Rabbit |
| PI3K | Cell signaling technology | 4257 | 1/1000 | Rabbit |
| p-Akt | Cell signaling technology | 4060 | 1/1000 | Rabbit |
| Akt | Cell signaling technology | 4691 | 1/1000 | Rabbit |
| p-mTOR | Cell signaling technology | 2971 | 1/1000 | Rabbit |
| mTOR | Cell signaling technology | 2972 | 1/1000 | Rabbit |
| p-p38 | Cell signaling technology | 4511 | 1/1000 | Rabbit |
| p38 | Cell signaling technology | 9212 | 1/1000 | Rabbit |
| p-MEK | Cell signaling technology | 9121 | 1/1000 | Rabbit |
| MEK | Cell signaling technology | 9122 | 1/1000 | Rabbit |
| p-ERK | Cell signaling technology | 4370 | 1/1000 | Rabbit |
| ERK | Cell signaling technology | 4695 | 1/1000 | Rabbit |
| p-GSK3β | Cell signaling technology | 9322 | 1/1000 | Rabbit |
| GSK3β | Santa Cruz | sc-9166 | 1/500 | Rabbit |
| GFAP | merck | 3140011 | 1/400 | Mouse |
| Iba-1 | Wako | 019-19741 | 1/400 | Rabbit |
| Goat anti-Rabbit | Santa Cruz | sc-2030 | 1/5000 | / |
| Goat anti-Mouse | Santa Cruz | sc-2031 | 1/5000 | / |
| Goat anti-Rabbit 488 | ThermoFisher | A-11008 | 1/500 | / |
| Goat anti-Mouse 488 | ThermoFisher | A-11001 | 1/500 | / |

Table 2. Detailed primers information.

| mnk1-Fd | 5'-CCATCGTGGATTCTGACAAGAG-3' |
| --- | --- |
| mnk1-Rv | 5'-GAACACTCGACTTCGACTGTG-3' |
| mnk2-Fd | 5'-TCGGGCTACTGACAGCTTCT-3' |
| mnk2-Rv | 5'-GACACAGGTCTGCACACGAG-3' |
| il-1β-Fd | 5’-ACCTTCCAGGATGAGGACATGA-3’ |
| il-1β-Rv | 5’-CTAATGGGAACGTCACACACCA-3’ |
| il-6-Fd | 5’-CACATGTTCTCTGGGAAATCG-3’ |
| il-6-Rv | 5’-TTGTATCTCTGGAAGTTTCAGATTGTT-3’ |
| tnf-α-Fd | 5’-GCCACCACGCTCTTCTGTCTAC-3’ |
| tnf-α-Rv | 5’-GGGTCTGGGCCATAGAACTGAT-3’ |
| il-10-Fd | 5’-CAGTACAGCCGGGAAGACAA-3’ |
| il-10-Rv | 5’-CCTGGGGCATCACTTCTACC-3’ |
| tgfβ-1-Fd | 5’-CCGCAACAAGCCATCTATG-3’ |
| tgfβ-1-Rv | 5’-TGCTTCCCGAATGTCTGACG-3’ |
| bdnf-Fd | 5’-AACCATAAGGACGCGGACTT-3’ |
| bdnf-Rv | 5’-TGCAGTCTTTTTATCTGCCG-3’ |
| trkb-Fd | 5’-CAGCACCAAGCAGCAAGAG-3’ |
| trkb-Rv | 5’-CAAGACCAGCAGGCATAAGC-3’ |
| gapdh-Fd | 5’- TGCACCACCAATTGCTTAGC-3’ |
| gapdh-Rv | 5’- GGCATGGACTGTGGTCATGAG-3’ |
